# Highly pathogenic avian influenza H5N1 virus infections of dairy cattle and livestock handlers in the United States of America

**DOI:** 10.1080/21505594.2024.2343931

**Published:** 2024-04-17

**Authors:** Hinh Ly

**Affiliations:** Department of Veterinary & Biomedical Sciences, College of Veterinary Medicine, University of Minnesota, Twin Cities, MN, USA

**Keywords:** HPAI H5N1, dairy cattle, cattle workers, poultry workers

Following a press release by the Minnesota Veterinary Medical Association (MVMA) on 26 March 2024 notifying its members of a juvenile goat on a Minnesota farm tested positive for the highly pathogenic avian influenza (HPAI) that was later confirmed to be H5N1 influenza virus by the National Veterinary Services Laboratories (NVSL) of the United States Department of Agriculture Animal and Plant Health Inspection Services (USDA-APHIS) [1], the MVMA reported two days later that some dairy cattle in Texas and Kansas states in the USA were tested positive for the same (H5N1) virus [2]. While the NVSL was working to confirm some presumptive positive test results from dairy herds in Texas, Idaho, and elsewhere [3], H5N1 virus were again detected in a dairy herd in New Mexico as well as in 5 additional dairy herds in Texas on 2 April 2024 [4]. It appears that this viral pathogen was spreading rapidly among dairy herds in several states in the USA. As of this writing (5 April 2024), H5N1 virus infections have been detected and confirmed in eight (08) dairy herds in Texas, three (03) in Kansas, two (02) in New Mexico, and one (01) herd each in Michigan and Ohio (Figure 1) [5]. It is important to note that H5N1 viruses found thus far [i.e. HPAI A(H5N1) viruses of the genetic clade 2.3.4.4b, which is the same virus clade found among birds globally] in different states appear to be very similar to the viral strain of the same genetic clade 2.3.4.4b originally identified in dairy cattle in Texas and Kansas where they were first reported on 25 March 2024. This is partly because some dairy farms in Michigan and Idaho have recently received (or imported) cattle from Texas with previously identified cases of H5N1 in their cattle herds [4].

**Figure 1.**
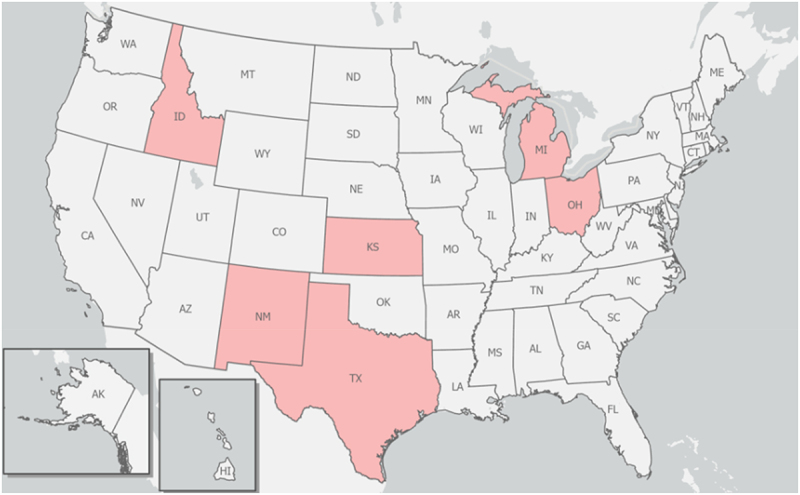
US jurisdictions with confirmed cases of HPAI H5N1 virus infections in dairy herds as of April 5, 2024 (Idaho, Michigan, Ohio, Kansas, New Mexico, and Texas shown in pink colour). It is noteworthy that a cattle worker in Texas and a poultry worker in Colorado were tested positive for H5N1 in 2024 and 2022, respectively. Graphic and data were derived and adapted from online sources (e.g. CDC and USDA-APHIS).

While it is known that influenza A viruses (IAV), which also includes H5N1, can frequently infect a variety of animal species [6,7], it is relatively rare to learn of cases of dairy cattle (or goat) being infected by the H5N1 virus. There have also been some anecdotal reports of H5N1 transmission between cattle in dairy herds, raising a concern that this virus could quickly adapt (for example, through viral genetic changes and/or genomic exchanges – antigenic drifts and/or shifts – although preliminary genetic evidence has suggested otherwise) to transmit among the same animal species (intraspecies transmission) and that it could also adapt to infect other species (interspecies transmission), including humans who have no pre-existing immunity against these new IAV strains that could result in excessive rates of mortality and morbidity in pandemic scenarios. There have been four significant IAV pandemics in modern human history that include the 1918–19 Spanish flu, the 1957 Asia flu, the 1968 Hong Kong flu, and the 2009 Swine flu. The 1918–19 Spanish flu caused an estimated 500 million infections and 50–100 million human deaths worldwide [6].

H5N1 virus infections have been frequently reported among wild birds in the USA and globally. However, they can cause outbreaks of infection in commercial and backyard poultry flocks [8] as well as sporadic infections of other animal species [9]. Zoonotic AIV infections usually occur through occasional virus transmissions from an avian or swine species to humans, but those types of an infection usually quickly resolve, and therefore, are self-limiting. However, certain types of IAVs, such as the avian H5 and H7 viruses can cause hundreds to thousands of infections with a high case fatality rate (30–50%) in humans [6]. Therefore, the possibility that some of these HPAI viruses (e.g. H5N1) can gain a foothold in human populations to mediate efficient human-to-human transmissions and potential pandemic scenario poses a significant public health risk. Notably, since the initial reports of H5N1 transmissions in dairy herds in several states in the USA [2,3], the US Centers for Disease Control and Prevention (CDC) issued a press release on 1 April 2024, to notify the public about 15 people with flu-like symptoms due to possible H5N1 exposure with dairy cattle have been tested with only one person, who had known direct exposure to a sick cow in a dairy herd in Texas, confirmed to be positive for H5N1 virus infection [10–12]. According to the CDC, this is the second person reported to have been tested positive for H5N1 virus infections in the USA with the first human H5N1 case occurring in 2022 in Colorado [13]. In that case, the infected person had a direct exposure to poultry during the depopulation (culling) of the poultry flock suspected of H5N1 virus infections. In both human cases, the patients reported relatively mild symptoms (i.e, tiredness in the Colorado case and conjunctivitis – eye redness due to inflammation – in the more recent case in Texas). Both patients were isolated, treated with the influenza antiviral drug oseltamivir (with the brand name Tamiflu), and recovered or is recovering [11,12]. While these US patients appeared to fare well, other human H5N1 infections have sometime resulted in severe illness (e.g. pneumonia) and/or death in other countries [6,14].

It is noteworthy that H5N1 virus infections of other farmed animals, such as the fur animals (mink) in Finland and Spain [15,16], commercial poultry flocks [8], as well as of domestic animals (cats or dogs) in the USA, France, Poland, and Italy [17–20] have recently been reported and summarized [7]. Since the infection and transmission routes of these highly pathogenic avian influenza viruses in farmed animals (e.g. dairy cattle) are still poorly understood, additional surveillance efforts and experimental research of H5N1 infection of the susceptible animal species, including cattle, are needed to fully understand its ecology, transmission route(s), and disease pathogenesis to prevent potential future outbreaks.

## Data Availability

No primary data is included in this article.
